# Assessing comparative asset-based measures of material wealth as predictors of physical growth and mortality

**DOI:** 10.1016/j.ssmph.2022.101065

**Published:** 2022-03-18

**Authors:** Katherine Woolard Mayfour, Daniel Hruschka

**Affiliations:** School of Human Evolution and Social Change, Arizona State University, Cady Mall, Tempe, AZ, 85281, USA

## Abstract

Social scientists and policymakers have increasingly relied on asset-based indices of household wealth to assess social disparities and to identify economically vulnerable populations in low- and middle-income countries. In the last decade, researchers have proposed a number of asset-based measures that permit global comparisons of household wealth across populations in different countries and over time. Each of these measures relies on different assumptions and indicators, and little is known about the relative performance of these measures in assessing disparities. In this study, we assess four comparative, asset-based measures of wealth—the Absolute Wealth Estimate (AWE), the International Wealth Index (IWI), the Comparative Wealth Index (CWI), and the “Standard of Living” portion of the Multi-Dimensional Poverty Index (MPI), along with a variable measuring television ownership—and compare how well each predicts health related variables such as women's BMI, children's height-for-age Z scores, and infant mortality at the household and survey level. Analyzing data from over 300 Demographic and Health surveys in 84 countries (n = 2,304,928 households), we found that AWE, IWI, CWI, MPI are all highly correlated (r = 0.7 to 0.9). However, IWI which is based on a common set of universally weighted indicators, typically best accounts for variation in all three health measures. We discuss the implications of these findings for choosing and interpreting these measures of wealth for different purposes.

## Highlights

• Assessing four comparative, asset-based measures of wealth.
• The four comparative asset-based measures of wealth are all highly correlated.
• International Wealth Index best accounts for variation in adult female BMI, child height-for-age, and infant mortality.
• Asset-based measures of wealth relying on universal indicators and weights perform better at explaining variance.

## 1 Introduction

Material wealth is a key factor shaping human behavior, psychology, and development and has shown well-established relationships with diet, fertility, physical growth, social behavior, cognitive function, and investment in education (Falkingham & Namazie, 2002; Godoy et al., 2010; Gwatkins et al., 2007; Hruschka et al., 2014; Kasper & Mulder, 2015; Montgomery, 2009; Newman & Thomson, 1989; Wagstaff, 2002; Ziker et al., 2016). For these reasons, social scientists have long been interested in the diverse ways that material wealth inequalities arise and persist, and how these inequalities shape human behavior, development, and health (Bowles et al., 2010; Cowell & Van Kerm, 2015; Deurzen et al., 2014; Douglas & Isherwood, 2002; Godoy et al., 2010, 2005; Green, 2015; Guedes et al., 2012; J.; Guyer, 1997; Murphy, 2014; Piketty, 2014; Sheppard et al., 2009; Smith et al., 2010; Undurraga et al., 2010; Pickett & Wilkinson, 2010; Zelner et al., 2012). In the domain of health specifically, social scientists have frequently shown that wealthier individuals tend to live longer, healthier lives (Halpenny et al., 2012; Hong et al., 2006; Jirapramukpitak et al., 2014; Pollack et al., 2007; Semyonov et al., 2013). These associations may arise because wealthier individuals can afford better nutrition, prevention services, and healthcare (Deaton, 2002). Alternatively, they may arise because health problems can lead to job loss or loss of savings to pay for medical bills (Olesen et al., 2013). Regardless of the causal direction, the associations illustrate the important reciprocal relationship between material wealth and health.

Material wealth is only one way social scientists have assessed socio-economic status of individuals and households. In high-income countries, economists, sociologists, and demographers have traditionally focused on income and expenditures, which can be difficult to measure in other contexts (Howe et al., 2012). Other common measures of socioeconomic status include education and occupation, which capture the social position and social resources of households and individuals (Kaiser et al., 2017; Maliti, 2019). Finally, social capital and related constructs, such as “wealth in people” and relational capital, are used to assess the social relationships that people can draw from to meet their needs (Coleman, 1994; Gurven et al., 2010; J. I.; Guyer, 1995; Mulder et al., 2009).

Among these diverse approaches to assessing socioeconomic status and well-being, material wealth is a useful measure of a household's ability to acquire desired goods and services, making measurement of wealth an important goal in the social sciences (Barrington-Leigh & Escande, 2018; Cowell & Van Kerm, 2015; Poirier et al., 2019; Pollack et al., 2007; Rutstein & Staveteig, 2014; Smits & Steendijk, 2015). It also serves as one key component of holistic measures of well-being such as the MPI (Alkire & Jahan, 2018). In low- and middle-income settings, measures of asset-based material wealth are particularly useful for reasons of cost, reliability, validity and stability (Howe et al., 2012; Jerven, 2013; Kaiser et al., 2017; Woolard et al., 2021). Thus, in these settings, researchers across a range of social sciences increasingly rely on asset-based indices of material wealth using ownership of durable goods, land, livestock, and access to basic services (Filmer & Pritchett, 2001; Howe et al., 2012; Hruschka et al., 2015; Rutstein et al., 2004; Rutstein & Staveteig, 2014; Smits & Steendijk, 2015; Woolard et al., 2021).

Asset-based wealth measures were originally developed as relative measures that permitted comparison within a single survey from a single country, but were not directly comparable across multiple countries or surveys and could not be used to track change over time (Rutstein & Staveteig, 2014; Smits & Steendijk, 2015). In the last decade, researchers have addressed the problem of relative wealth levels by creating wealth indices that can be compared both within and across survey samples (Harttgen & Vollmer, 2013; Howe et al., 2012; Hruschka et al., 2015; Kaiser et al., 2017). These include the International Wealth Index (IWI) (Smits & Steendijk, 2015), the Comparative Wealth Index (CWI) (Rutstein & Staveteig, 2014), the Absolute Wealth Index (AWE) (Hruschka et al., 2015), and the standards of living component of the Multi-Dimensional Poverty Index (MPI) (Alkire & Santos, 2010). In the next section, we outline the methodology for each of these wealth measures, as well as the implicit assumptions underlying the calculation of each one.

### 1.1 Comparative asset-based wealth Measures

Each of the following indices were developed to provide asset-based measures of wealth that were fully comparable across countries and timepoints. Here we outline key differences between them. More information on calculation of each measure can be found in the supplemental materials.

#### 1.1.1 International Wealth Index

The International Wealth Index (IWI) was developed by applying principal components analysis (PCA) on 12 common assets and indicators to a pooled database of 165 household surveys from 1996 to 2011 in 97 low-and middle-income countries, covering 2.1 million households in total (Smits & Steendijk, 2015). The database that was used consisted mainly of DHS surveys, UNICEF's Multiple Indicator Clusters Surveys (MICS), supplemented with some IPUMS census survey datasets and a number of stand-alone surveys (Smits & Steendijk, 2015). The PCA analysis resulted in a raw score that was subsequently rescaled to 0–100. The IWI was tested and validated against removal of assets, countries, time periods, and correlations with other welfare and poverty measures (Smits & Steendijk, 2015). Although the indicators and weightings used by IWI are universal, it is important to note that it includes two items (“cheap utensils” and “expensive utensils”) that can accommodate survey-specific items that are valued either below 50 USD or above 250 USD (see Supplementary Materials).

#### 1.1.2 Multi-Dimensional Poverty Index

The Multi-Dimensional Poverty Index was developed in order to address “failures in functionings” that other wealth scores may not capture (Alkire & Santos, 2010). It is unique from the other methods because it attempts to capture these failings across three separate dimensions, measuring health (including child mortality and nutrition), education (including years of schooling and child enrollment), and standard of living (including electricity, drinking water, sanitation, housing materials, cooking fuel, and a selection of assets). Specific dimensions and indicator variables were chosen to align with the Millennium Development Goals (MDGs), and later the SDGs (Alkire & Jahan, 2018; Alkire & Santos, 2010). For this paper, we will focus on the MPI Standard of Living score (MPI-SL) which most closely aligns with other asset-based measures of household material wealth (see Supplementary Materials). The MPI has gone through several changes and iterations since it was developed. The version of the MPI we use was released in 2018, and draws on the MPI versions from 2010 to 2014 while aligning the MPI with the new SDGs to best measure key areas of deprivation (Alkire & Jahan, 2018). The most current version of the MPI was released in 2020, and relies on virtually the same variables as the 2018 version (Oxford Poverty & Human, 2020).

#### 1.1.3 Comparative Wealth Index

The CWI was derived from an existing relative wealth measure, the DHS Wealth Index, which in turn relies on survey-specific assets and asset weights derived from a survey-specific PCA (Rutstein & Staveteig, 2014). To render these survey-specific measures comparable, the CWI adjusts the DHS Wealth Index for each specific survey relative to a “baseline survey” which was selected to have median survey characteristics (e.g. Gross National Income per capita and survey year). Vietnam 2005 was chosen as the baseline survey for the original estimation. DHS Wealth indices from different surveys are then made comparable with the baseline survey's index by using eight anchoring values: the average wealth of households that had one, two, three, or four Unsatisfied Basic Needs (UBNs)—inadequate walls or floors, crowding, inadequate sanitation, and high economic dependence—and the wealth at which half of all households owned each of four consumer goods—TV, car/truck, refrigerator, landline telephone (see Supplementary Materials). This involved fitting a regression that predicted wealth values for a specific survey by the baseline survey's wealth values. The slope and intercepts from this regression were then applied to that survey's DHS wealth index to calculate the CWI. The CWI was validated using sensitivity testing for removal of or edits to certain cutpoints, as well as the impact of using different baseline surveys and changing the measurement of UBNs. Because of the CWI's reliance on the DHS Wealth Index, we did not include the DHS Index as a separate measure, since it would explain the same within-country variation as the CWI, and no between-country variation.

#### 1.1.4 Absolute Wealth Estimate

The AWE was also derived from the DHS Wealth Index, relying on the relative rank of households on the Wealth Index and the estimated shape of the wealth distribution among all surveyed households (Hruschka et al., 2015). The shape of the wealth distribution for each country and survey year was calculated using three parameters: the mean wealth per capita for that country in a given year, the Gini coefficient (a measure of wealth variance and inequality) for that country, and the best combination of the Pareto and log-normal distributions to achieve optimal skewness of the wealth distribution (Hruschka et al., 2015). With this, it was possible to estimate the absolute wealth of each household by mapping their DHS Wealth Index ranking onto the shape of the overall wealth distribution (see Supplementary Materials). These AWE scores were then validated against the World Bank's poverty headcount, along with several markers of nutritional status, including women's BMI and children's height-for-weight and height-for-age Z scores.

### 1.2 Comparison

IWI, MPI-SL, CWI, and AWE are similar in many key areas. They all rely on assets and access to services as proxies for household economic well-being. They also aim to provide a comparable measure of household material wealth across multiple timepoints and countries.

However, these methods also vary in several key areas, including underlying assumptions about the level of context-dependence, choice of asset and indicators (See Table 1), and assignment of weights to assets and indicators (see Table 2). The International Wealth Index and Multi-Dimensional Poverty Index Standard of Living Score are completely independent of context, relying on a universal list of assets and weightings for those assets to assign household wealth values. The IWI derived asset weights empirically from a PCA applied to 165 surveys. The MPI-SL derived indicators from Sustainable Development Goal, and it weights the assets equally. The Comparative Wealth Index differs from the MPI-SL and IWI, because it incorporates a measure of wealth, the DHS Wealth Index, that uses survey-specific assets and weights. That said, it does rely on a common set of anchors—based on unmet basic needs—to scale survey-specific DHS Wealth Indices to be comparable with a common baseline survey. Finally, the Absolute Wealth Estimate is also context dependent, in that it relies on survey-specific assets and weights to rank households. Like the CWI, it relies on the DHS Wealth Index, but it then maps each household onto a wealth distribution for that country based on its GDP per capita and GINI measures of wealth inequality. Finally, the AWE presents scores in a metric of International Dollars, which can be easily compared with other economic metrics.

**Table 1:** Assumptions and key indicators underlying four asset-based wealth measures.

Method:	International Wealth Index (IWI)	MPI Measure of Standard of Living (MPI-SL)	Comparative Wealth Index (CWI)	Absolute Wealth Estimate (AWE)
Assumptions	- Universal set of assets - Universal asset weights	- Universal set of indicators based on SDGs - All assets weighted equally	- Survey-specific assets - Survey-specific asset weights - Anchored by universal basic needs	- Survey-specific assets - Survey-specific asset weights - National wealth distribution
Indicators	12 indicators, 16 levels	6 indicators and levels	Variable indicators, 8 anchors	Variable indicators
Strengths	- Relies on only 12 indicators available in many surveys. - Easy to calculate	- Relies on only 6 indicators available in many surveys. - Easy to calculate	- Requires two sets of inputs: DHS Wealth Index and UBNs	- Requires three inputs: DHS Wealth Index, wealth per capita, GINI - Broadly comparable units
Weaknesses	- Does not adjust for new surveys - Can't be generalized historically	- Updated frequently, rendering old versions not comparable	- Only comparable across studies if the same baseline is selected	- Relies on assumed wealth distribution

**Table 2:** Assets, anchors and scoring underlying four asset-based wealth measures.

	International Wealth Index (IWI)	MPI Measure of Standard of Living (MPI-SL)	Comparative Wealth Index (CWI)	Absolute Wealth Estimate (AWE)
Assets	Television Refrigerator Phone Bicycle Car Cheap utensils (<$50 USD), Expensive utensils (>$250 USD) Electricity # of sleeping rooms Quality of drinking water Quality of toilet Quality of floor	Radio Television Telephone Computer Animal cart Bicycle Motorcycle Refrigerator Car/Truck Sanitation quality Drinking water quality Electricity Housing materials	DHS Wealth Factor Score (Context dependent for each country)	DHS Wealth Factor Score (Context dependent for each country)
Anchors	None	None	Floor & Wall quality Crowding Sanitation quality Economic dependency TV, Car/Truck, Landline Telephone, Refrigerator	None
Scoring	Assets and indicators weighted by PCA, score ranges from 0–100	All assets and indicators weighted equally	Assets and indicators weighted with PCA for each country UBNs used as anchors, score is measured as standard deviations from baseline survey.	Assets and indicators weighted with PCA for each country Households plotted onto assumed wealth distribution Score converted into International Dollars

These four asset-based wealth measures all aim to compare material wealth across individuals and households. However, they make crucially different assumptions about which assets and indicators are included and how they should be weighted. Some are based on a universal set of items and weights (IWI, MPI), while others use context-specific items and weights (IWI, CWI). Each relies on a different number of assets, ranging from 6 universal for the MPI to more than 100 country-specific items for measures based on the DHS Wealth Index. This leaves the researcher with a range of choices with little information about the relative ability of these different indices to predict outcomes of interest.

In this paper, we compare the relative performance of four comparative asset-based methods in predicting several health outcomes which have traditionally been associated with material wealth. Analyzing data from over 300 Demographic and Health Surveys (DHS) in 84 countries, we assess: (1) how these measures correlate with each other, and (2) how well they explain variance in three measures of physical growth (adult women's BMI, child height-for-weight z scores) and mortality (infant mortality) both within- and between-countries.

## 2 Methods

### 2.1 Sample

We used data from the Demographic and Health Surveys (ICF International) including over 2.3 million households in 84 low- or middle-income countries, collected between 1992 and 2014 that had estimates for all four comparative wealth indices.

For BMI, we analyzed data from 281,173 females ages 40–49 who were not currently pregnant. We focused on female data since significantly less data has been collected on men in the DHS surveys. The age range of 40–49 was selected because this age group generally shows the strongest relationship between household wealth and BMI (Hruschka et al., 2014). For children's height-for-age Z scores, we analyzed 401,398 children ages 12–36 months old. This age range was selected because (1) previous research has shown that HAZ might be an unreliable measure of growth and nutrition for children under one year old, and (2) some DHS surveys only collect height up to 36 months of age (Hackman & Hruschka, 2018). For infant mortality analyses, we focus on survival between 0 and 11 months among the most recent born children (between 12 and 47 months of age) of sampled women (n = 74,492).

### 2.2 Measures

#### 2.2.1 Outcomes

Based on their well-established theoretical and empirical associations with material wealth, we chose two measures of physical growth as well as infant mortality as benchmarks for assessing the predictive ability of each of the four asset-based wealth measures.

##### **2.2.1.1** BMI (individual and survey-level)

In low- and middle-income countries, greater BMI is usually associated with higher absolute wealth and higher socioeconomic status (Hruschka, 2017, p. 201; Hruschka & Brewis, 2013; Sobal & Stunkard, 1989). BMI was calculated from the DHS collected measures of weight (in kg) and height (in meters). We then calculated BMI as weight (kg)/height (m)2, and treated it as a continuous variable. Biologically implausible BMI values above 90 or less than 10 were excluded from analysis which is a slightly more liberal cutoff than used in previous studies (Li et al., 2009).

##### **2.2.1.2** Height-for-age z-scores (individual and survey-level)

Children's height-for-age z-scores were selected as a benchmark due to their well-established relationships with wealth in low-and middle-income countries (Hackman & Hruschka, 2020; Hong et al., 2006; Hong & Mishra, 2006). Height and age in months were collected by DHS fieldworkers. We calculated height-for-age z-scores according to the current World Health Organization reference categories (WHO Multicentre Growth Reference Study Group, 2006), and excluded biologically implausible cases that were more than ± 6 SD (Hackman & Hruschka, 2020). HAZ scores were also treated as a continuous variable.

##### **2.2.1.3** Infant mortality (survey-level)

Infant mortality was selected as a benchmark because it is known to be greater in poorer households (Deurzen et al., 2014; Moser et al., 2005; Sastry, 2004)**.** Information on infant mortality was estimated from in-depth birth histories for women sampled by the DHS surveys. We then created an infant mortality variable that represents whether a woman's most recently born child died between 0 and 11 months from birth. The sample was limited to the most recently born child in the past 12–47 months since the interview, so that there is only one birth considered per woman, the infant has been observed for at least one year, and the final observation is no more than five years from the interview. The outcome is the proportion of eligible children who died between 0 and 11 months from birth.

#### 2.2.2 Comparative asset-based wealth measures

For each survey, we calculated IWI, CWI, AWE, and MPI scores. We then calculated a categorical version of each index in order to better graphically represent the relationship between all wealth indexes and the measures of physical growth and mortality.

##### **2.2.2**.**1** International Wealth Index

IWI estimates were made available by the Global Data Lab (www.globaldatalab.org). For comparative figures, we also created a categorical variable for the IWI using increments of 5 to create 20 separate categories.

##### **2.2.2.2** Comparative Wealth Index

CWI scores were generated using the regression coefficients calculated according to the approach outlined in (Rutstein & Staveteig, 2014), which were then multiplied with the DHS wealth factor score for each household. For comparative figures, we also created a categorical version of the CWI by increasing in increments of 0.19 to create 20 categories (with the highest category as > 1.87 and the lowest category as < −1.76).

##### **2.2.2.3** Absolute Wealth Estimates

AWE scores were calculated according to the procedures laid out in (Hruschka et al., 2015). For comparative figures, AWE was also binned into 19 separate categories each representing an approximately 50% increase in household wealth per capita (in International dollars: cutpoints at roughly <90, 135, 200, 300, 450, 680, 1030, 1540, 2300, 3500, 5200, 7800, 11700, 17500, 26300, 39400, 59100,> 88600).

##### **2.2.2.4** MPI standard of Living Score

Code to calculate the MPI-SL was developed according to the guidelines outlined by (Alkire & Santos, 2010) and with the updates from (Alkire & Jahan, 2018). Sanitation quality, drinking quality, and housing quality were judged as deprived or improved according to guidelines outlined in MPI reports. In cases of confusion about water or sanitation quality we referred to WHO guidelines. Because of the nature of the MPI-SL, it had to be reverse coded and scaled from 0 to 100 in order to be easily comparable with the other measures. MPI-SL is not continuous but naturally categorical with 5 values.

##### **2.2.2.5** Television

To compare the four measures with a single-asset measure, we also considered a variable for television ownership, an asset that is ubiquitous across countries, varies within and between countries, and is included in many of the calculations of the other measures.

We used a variable collected by the DHS that indicated if there was a television present in the household or not.

#### 2.2.3 Variables for exploratory analyses

##### **2.2.3.1** GDP

Given that some comparative wealth measures depend on GDP per capita to estimate between country differences (Harttgen & Vollmer, 2013; Hruschka et al., 2015), we also examined the performance of the natural log of World Bank estimates of GDP per capita in international dollars (constant 2011 equivalent, PPP) in predicting physical growth and infant mortality. We used log estimates of GDP in order to most accurately match the methods used by the wealth measures that rely on GDP, since they use logged values in order to address skew in wealth distribution.

#### 2.2.4 Covariates for individual-level analyses

Several variables were used as covariates for the individual-level analyses: the woman or mother's age, education, and parity, and the survey year (centered at 2000). Women's age was treated as categorical with each year as a category, in order to understand any relationships that can't be captured using linear or quadratic functions. Although there is some evidence of non-linearity at higher educational levels, education often has a positive relationship with BMI in low- and middle-income countries (Hruschka et al., 2019; Mazariegos et al., 2021; Ozodiegwu et al., 2020). This variable was dummy-coded to represent years of education (none, primary, secondary, and post-secondary) (Frost et al., 2005; Rodolfo et al., 2000). Primary education was chosen as the reference category in order to represent the most typical amount of education received. Parity was top-coded at ten, and the reference category was set at parity of two. Survey year was also included as a measure of other improvements that occurred in a country independent of increases in wealth (centered at 2000). **BMI:** Since women's age was restricted to 40–49 y for these analyses, and we used 49 as a reference category. **Height-for-age z-scores:** In these analyses, we used 25 years as a reference category for mother's age, to represent a mother in the middle of her reproductive years. Additionally, we controlled for children's age (centered at 24 months) as this was the middle of the age range, and child's sex as a dichotomous variable where 1 = male, 2 = female (reference category = female).

### 2.3 Analysis

#### 2.3.1 How well do each of these measures of household wealth correlate with each other?—

We estimated Pearson's correlation coefficient on 2,304,928 households among the four asset-based wealth measures—IWI, CWI, AWE, and MPI. We also plotted the mean household values of each wealth measure across the categorical form of each other wealth measure to visually represent the shape of the relationship between variables.

#### 2.3.2 How well do each of these measures of household wealth explain within and between-country variation in women's BMI and child height-for-age z (HAZ) scores?

We used mixed effects linear regression models with country-level random effects for intercept and year of survey to assess the proportion of variance in BMI and HAZ scores accounted for by each of the four asset-based wealth measures—IWI, CWI, AWE, and MPI, along with the variable of TV ownership. We also included the covariates outlined above in the model (see section 2.2.4). Due to collinearity of the main wealth measures, we conducted independent regressions for each of the five wealth measures as a predictor. We then calculated the proportion of variance explained in these two growth outcomes relative to a null model including all covariates except for the wealth measure, which follows a precedent set by previous research studying indicators (Lloyd & Auld, n.d.; Michalos, 2004). We calculated $R^{2}$ for each wealth measure for the within country variation *(1-(model residual/null model residual))* and the between country variation *(1-(variance in random intercept/variance in random intercept)).* We also plotted the values of BMI and HAZ for each category of the categorical wealth variables to further examine their relationship. Analyses were run in IBM SPSS Statistics 25.

#### **2.3.3** How well do survey-level means in these four wealth indices predict women's BMI, children's height-for-age z-scores, and infant mortality

Next, we examined graphically how each wealth measure related to BMI, height-for-age z-scores, and infant mortality at a survey level by using scatterplots to compare survey-level means. Analyses were run in R Studio.

#### 2.3.4 Exploratory analyses of survey-level GDP per capita

In order to better understand the relatively poorer performance of the AWE at accounting for between-country variation in key outcomes, we also analyzed the survey-level performance of GDP per capita at explaining variance in BMI, height-for-age z-scores, and infant mortality, since AWE estimates are based on GDP per capita. We use R^2^ of the bivariate association between ln(GDP per capita) and these measures. Analyses were run in R Studio.

## 3 Results

### **3.1** How correlated are the four asset-based measures of household wealth?

All measures were highly correlated (see Table 3). The highest correlations were between the IWI and CWI (0.90) and IWI and MPI-SL (0.87), as well as the CWI and AWE (0.86). The lowest correlations were between the MPI-SL and the AWE (0.79), and all measures with the television variable.

**Table 3:** **Correlations Between Measures (all significant at the 0.01 level).** IWI = International Wealth Index, MPI-SL = Multi-dimensional Poverty Index Standard of Living Scale, CWI = Comparative Wealth Index, AWE = Absolute Wealth Estimate, TV = has television.

	IWI	MPI-SL	CWI	AWE
MPI-SL	.89			
CWI	.90	.84		
AWE	.83	.79	.86	
TV	.80	.73	.72	.66

### 3.2 How well do each of each of these measures explain within and between country variance in physical growth?

#### 3.2.1 Body mass index

**Fig. 1:**
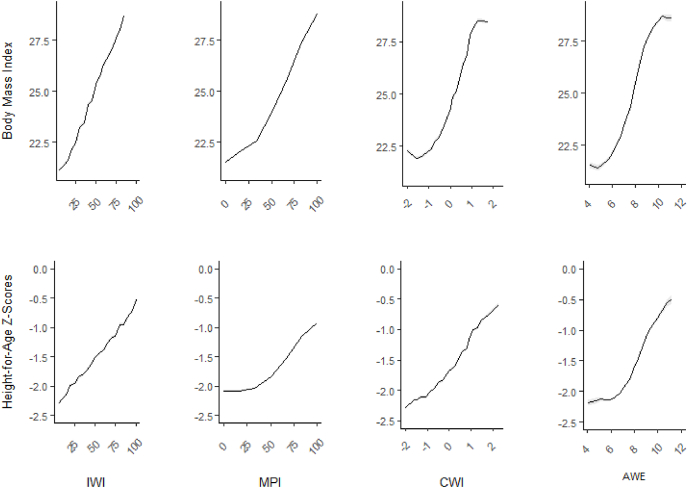
Mean women's BMI and children's HAZ at different levels of the four comparative wealth indices.

After controlling for key covariates, all four measures performed comparably in explaining within country variation in women's BMI (R^2^ = 0.04 to 0.07), (Table 4, Fig. 1). The IWI best explained between country variation in women's BMI (R^2^ = 0.45), followed by the MPI-SL (R^2^ = 0.41), the CWI (R^2^ = 0.33), the TV ownership variable (R^2^ = 0.29), and finally the AWE (R^2^ = 0.17) (Table 4, Fig. 1).

**Table 4:** Proportion of variance explained (R^2^) between and within countries in women's BMI, children's HAZ by IWI, MPI, CWI, AWE, and TV Ownership.

Measure	Level	International Wealth Index (IWI)	Multi-Dimensional Poverty Index Standard of Living (MPI-SL)	Comparative Wealth Index (CWI)	Absolute Wealth Estimate (AWE)	Television Ownership
**BMI** **(N =** 281,173)	Within country	0.07	0.06	0.07	0.07	0.04
	Between country	0.45	0.41	0.33	0.17	0.29
**HAZ** **(N =** 401,398)	Within country	0.02	0.01	0.02	0.02	0.01
	Between country	0.38	0.33	0.32	0.14	0.20

#### 3.2.2 Height-for-age z-scores

None of the measures explained substantial portions of within-country variance children's HAZ (AWE R^2^ = 0.017, IWI R^2^ = 0.016, CWI R^2^ = 0.015, MPI-SL R^2^ = 0.013, TV ownership R^2^ = 0.008). The IWI best explained between country variation in children's HAZ (R^2^ = 0.37), followed by the MPI and CWI (both R^2^ = 0.32), TV ownership (R^2^ = 0.20), and the AWE (R^2^ = 0.14).

### 3.3 Survey-level associations

**Fig. 2:**
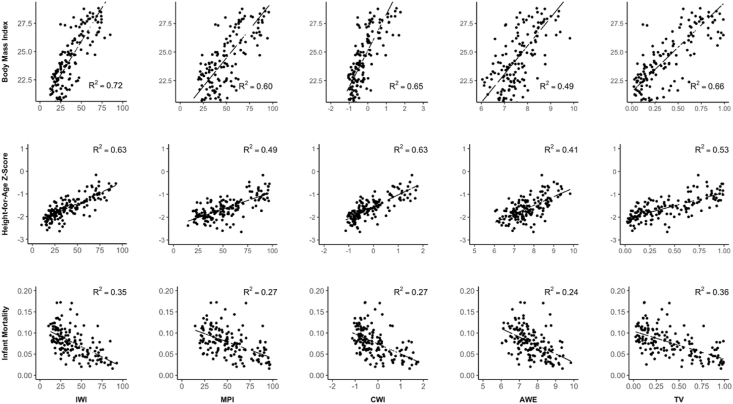
Relationship between survey-level wealth measures and survey-level measures of physical growth and infant mortality.

Survey-level means of IWI outperformed the other measures in explaining the survey-level variation in all three indicators (R^2^ for BMI = 0.72; R^2^ for HAZ = 064; R^2^ for infant mortality = 0.32; Fig. 2).

### 3.4 Exploratory GDP analyses

Among the four wealth indices, AWE performed the worst at accounting for between-survey variation in women's BMI, children's height-for-age, and infant mortality when compared to the other wealth indices (Fig. 2). GDP per capita (logged), which is the basis for calibrating AWE across countries, also performed relatively poorly at accounting for between-survey variation in BMI (R2 = 0.54), height-for-age z-scores (R2 = 0.42), and infant mortality (R2 = 0.27).

## 4 Discussion

While all four asset-based indices for household wealth—the International Wealth Index, the Comparative Wealth Index, the Absolute Wealth Estimate, and the Standard of Living section of the Multi-Dimensional Poverty Index—along with the TV ownership index were highly correlated, the IWI best explained between country and survey variation in all three of the health-related benchmarks: women's BMI, child height-for-age Z-scores, and risk of infant mortality. These findings suggest that the IWI could serve as a useful benchmark for comparison of future wealth indexes, such that any new proposed indexes must explain variance over and above the IWI.

The two measures that best explained between-country variation in the three benchmarks—the IWI and the MPI-SL—both relied on a universal set of indicators and universal weights for those indicators. One possible reason for their better performance is that people may exhibit relatively universal preferences for and also bear relatively uniform costs for these items across diverse settings. The better performance of measures with universal indicators and weights merits further investigation. The poor performance of GDP per capita at explaining between-survey variation in benchmarks likely accounts for the particularly poor performance of AWE—which relies specifically on GDP per capita for calibrating between-survey differences. This suggests that any measure relying on GDP per capita for this purpose (Fink et al., 2017; Harttgen & Vollmer, 2013) will likely perform worse than measures such as IWI and MPI-SL that rely instead on differences in asset ownership to estimate between-country differences.

Fig. 2 also identifies non-linearities in the association between wealth and health for all asset-based measures except for IWI. This may be due to truncation of the measures at very high and low levels of wealth, and further research should examine the reasons for these non-linearities.

Measures that use fewer indicators may perform worse at explaining within-country variation, and may produce less reliable estimates at the individual or household level. The exploratory analyses of a single TV indicator illustrate this point at the extreme. Specifically, the single TV item performs very poorly at accounting for within-country analyses, but at the aggregate level of surveys and countries it performs quite well.

These preliminary analyses suggest a few additional lines of inquiry. First, the current analyses only considered DHS data, and future work should investigate these and other wealth indexes on other survey types, such as the Multiple Indicator Cluster Surveys and World Health Surveys (Aryeetey et al., 2010; Balen et al., 2010; Poirier et al., 2019). Additionally, this study does not consider other measures, such as the Poverty Probability Index (PPI). Originally the Simple Poverty Scorecard (*PPI*, n.d.; *Simple Poverty Scorecard*, n.d.), the PPI is designed specifically for each country with a range of indicators that may not be available in most demographic surveys. This presents a problem for comparability using large datasets such as the DHS, since many of the assets and indicators used for the Poverty Probability Index are so specific that this information is not collected by the DHS. One potential way of testing the association could be to isolate PPI surveys that have assets and indicators available in DHS surveys, and then compare them with the IWI, CWI, AWE, and MPI-SL scores for the country. Finally, the current study assessed the performance of these asset-based wealth indices as predictors of health. Future work may consider additional comparisons, such as concentration indices of health compared with concentration indices of wealth as indicators of relative inequality across populations (Koolman & Van Doorslaer, 2004).

Here, we explore indices that largely capture material wealth gained through market-based economies. In recent years, researchers have proposed alternative multidimensional indices that capture diverse livelihoods that go beyond success in market economies. Additional work should consider the performance of these alternative indices (Ferreira & Lugo, 2013; Hackman et al., 2020; Hadley et al., 2019; Hargreaves et al., 2007; Hruschka et al., 2017; Klasen & Blades, 2013; Lachaud et al., 2020). Additionally, some recent research has suggested the benefits of using wealth indices to measure context-specific wealth across the lifecourse, which, when consistently measured, could help to reveal differences in wealth patterns across countries (Varghese et al., 2021). Other research has suggested that assets used to form the DHS wealth index may better reflect overall SES, but certain indicators stand out as predictors of positive child health (Karlsson et al., 2020).

While measures using a universal set of assets (i.e., IWI, MPI) had better overall performance in the samples considered here, one weakness with such measures is that they rely on assets and indicators that may not be useful for making longer-term historical comparisons (e.g., populations prior to the availability of televisions) and may not be able discriminate between households within small-scale societies. For instance, the Tsimane of lowland Bolivia have very low rates of ownership of most items used in the IWI or MPI. This may mean that there are effectively few differences in material wealth between households or rather that we must turn to other assets to understand such differences (Godoy et al., 2005; Varghese et al., 2021).

Despite these caveats, we find that an asset-based measure of material wealth relying on a universal set of indicators and weightings performs better than other measures at accounting for both within-country variation in physical growth as well as between-survey variation in physical growth and infant mortality. Future research that investigates why the IWI outperforms other wealth indexes is needed to be able to construct even better asset-based measures of wealth. This is especially relevant as more indices move towards country-specific measures of poverty, such as the PPI. Future research would ideally also examine which measures are most sensitive to wealth extremes, and make recommendations for measurements that best differentiate among the poorest members of the wealth distribution.

## Author statement

Kate Woolard Mayfour: Conceptualization, Software, Formal Analysis, Investigation, Data Curation, Writing – Original Draft, Visualization.

Daniel Hruschka: Conceptualization, Methodology, Software, Formal Analysis, Resources, Data Curation, Writing – Review & Editing, Supervision, Administration, Funding Acquisition.

## Funding

This work was supported by the 10.13039/100000001National Science Foundation (BCS-1150813) awarded to Dr. Daniel Hruschka, and by the 10.13039/100000001National Science Foundation (BCS-1658766) awarded to Dr. Daniel Hruschka.

## Financial interest

None.

## Declaration of competing interest

None.
