# Supplementary material for: Assessing comparative asset-based measures of material wealth as predictors of physical growth and mortality
Source: SSM Popul Health. 2022 Mar 18;17:101065. doi: 10.1016/j.ssmph.2022.101065 (PMC8956810; doi:10.1016/j.ssmph.2022.101065)
Supplement: Multimedia component 1 [file mmc1.docx]

**Supplement A: Information about the Construction of the Wealth Indices**

**IWI:** The IWI relies on 12 common assets and indicators, some with many levels. These are: the presence in the household of a television, refrigerator, phone, car, bicycle, cheap utensil or expensive utensil, type of flooring (three levels: high, medium, and low) toilet type (three levels: high, medium, and low), number of rooms (three levels: zero to one, two, three or more), access to electricity, and water source quality (high, medium, and low). which were originally derived by applying principal components analysis (PCA) to a pooled database of 165 household surveys from 1996 to 2011 in 97 low-and middle-income countries, covering 2.1 million households in total (Smits & Steendijk, 2015). We calculated the IWI according to guidelines from the Global Data Lab (Smits & Steendijk, 2015), and also created a categorical version of the IWI that consisted of 20 categories moving up in 5-point increments, where scores 0-5 would be binned as a “1”, 5-10 would be binned as a “2”, etc.

**MPI-SL:** The MPI was created to align with the Millennium Development Goals, and later the Sustainable Development Goals (Alkire & Jahan, 2018a; Alkire & Santos, 2010). The MPI measures three dimensions of wellbeing, including health, education, and standard of living (Alkire & Santos, 2010), however following previous research, we will use only the MPI Standard of Living score (MPI-SL) which is most easily comparable to other wealth indices (Woolard et al., 2020). The MPI-SL measures whether or not a household is deprived, and relies on six indicators: (1) quality of cooking fuel commonly used, (2) sanitation quality, (3) drinking water quality, (4) access to electricity, (5) quality of housing materials (roof, wall, or floor) and (6) possession of more than one of any of 10 assets (radio, TV, telephone, computer, animal cart, bicycle, motorbike, refrigerator, car, and truck). Unlike the IWI, the MPI-SL does not have any additional levels. MPI-SL was calculated by following guidelines outlined in (Alkire & Santos, 2010) and the updates in (Alkire & Jahan, 2018b). The MPI-SL is already categorical because there are only seven possible scores (from deprivation in no categories to deprivation in all categories), so we did not create a categorical version.

**CWI:** The Comparative Wealth Index creates a generalizable measure of wealth using DHS survey data. The CWI is based on an existing wealth measure, the DHS Wealth Index, which is a survey-specific (completely context dependent) measure of wealth (Rutstein & Staveteig, 2014). The CWI relies on the use of a “baseline survey” that has a median GNI-PPP (Gross National Income per capita based on Purchasing Power Parity, a measure of household monetary income that is comparable across place and time), and is in a median DHS year. The procedure involves eight anchors based on four consumer goods—ownership of a car/truck, refrigerator, fixed telephone, and television—and the cumulative total of four unmet basic needs—more than three people per sleeping room, inadequate toilet or drinking water facilities, and households with more than three household members per worker. For each survey and each anchor, a logistic regression predicted the DHS wealth index cutpoint at which 50% of households scored “yes” on that anchor. Then a regression predicting the baseline survey’s 8 cutpoints from the target survey’s 8 cutpoints provided regression coefficients for adjusting the target country households’ DHS wealth indices to be comparable with the baseline country’s DHS wealth indices. We used these publicly available regression coefficients to calculate the CWI from each survey’s DHS wealth index.

**AWE:** AWE calculates the shape of the wealth distribution for each country and survey year using: (1) mean wealth per capita in a country in a given year, (2) a measure of wealth variance and inequality (the Gini), and (3) the best combination of Pareto and log-normal distributions to achieve optimal skewness of the wealth distribution (Hruschka et al., 2015). Next, households are mapped onto the shape of the overall wealth index based on their DHS Wealth ranking. This produces an estimate of absolute wealth for each household. We also created a categorical version of the AWE which was binned into 19 separate categories with each representing an approximately 50% increase in household wealth per capita (in International dollars: cutpoints at roughly <90, 135, 200, 300, 450, 680, 1030, 1540, 2300, 3500, 5200, 7800, 11700, 17500, 26300, 39400, 59100, > 88600).
